# Temporal and Spatial Distribution of the Microbial Community of Winogradsky Columns

**DOI:** 10.1371/journal.pone.0134588

**Published:** 2015-08-06

**Authors:** David J Esteban, Bledi Hysa, Casey Bartow-McKenney

**Affiliations:** Department of Biology, Vassar College, Poughkeepsie, New York, United States of America; New York University School of Medicine, UNITED STATES

## Abstract

Winogradsky columns are model microbial ecosystems prepared by adding pond sediment to a clear cylinder with additional supplements and incubated with light. Environmental gradients develop within the column creating diverse niches that allow enrichment of specific bacteria. The enrichment culture can be used to study soil and sediment microbial community structure and function. In this study we used a 16S rRNA gene survey to characterize the microbial community dynamics during Winogradsky column development to determine the rate and extent of change from the source sediment community. Over a period of 60 days, the microbial community changed from the founding pond sediment population: Cyanobacteria, Chloroflexi, Nitrospirae, and Planctomycetes increased in relative abundance over time, while most Proteobacteria decreased in relative abundance. A unique, light-dependent surface biofilm community formed by 60 days that was less diverse and dominated by a few highly abundant bacteria. 67–72% of the surface community was comprised of highly enriched taxa that were rare in the source pond sediment, including the Cyanobacteria *Anabaena*, a member of the Gemmatimonadetes phylum, and a member of the Chloroflexi class Anaerolinea. This indicates that rare taxa can become abundant under appropriate environmental conditions and supports the hypothesis that rare taxa serve as a microbial seed bank. We also present preliminary findings that suggest that bacteriophages may be active in the Winogradsky community. The dynamics of certain taxa, most notably the Cyanobacteria, showed a bloom-and-decline pattern, consistent with bacteriophage predation as predicted in the kill-the-winner hypothesis. Time-lapse photography also supported the possibility of bacteriophage activity, revealing a pattern of colony clearance similar to formation of viral plaques. The Winogradsky column, a technique developed early in the history of microbial ecology to enrich soil microbes, may therefore be a useful model system to investigate both microbial and viral ecology.

## Introduction

Sediments and soils are known to contain extraordinary diversity and abundance of microorganisms and a significant amount of research is being done to investigate the factors that influence and maintain such high microbial diversity [1]. The structure of a microbial community is the result of environmental factors, evolutionary processes, and neutral or stochastic processes [2–5]. Recent studies have emphasized the importance of local conditions and environmental gradients in structuring microbial communities such as in shallow lakes [6], microbial mats [7] and rice paddy soils [8,9]. Environmental variation caused by seasonal change has been shown to influence community structure in sediment communities [10] and drive a cyclic pattern of community turnover in some ocean communities [11,12], while others are seasonally stable [13].

Given the high abundance and diversity of bacteriophages in all tested environments [14–17], it is likely that bacteriophages also play a key role in microbial community structure, dynamics, and function [18]. Indeed, in marine microbial communities, viruses have been shown to drive major shifts in abundance and structure and to exert control over bacterial diversity [19–21]. Research on the diversity and role of phages in soil and sediment microbial communities lags behind that of marine environments despite phage abundance in these environments being typically higher than aquatic environments [22].

Winogradsky columns are enrichment cultures, typically made by filling transparent cylinders with soil or sediment and incubating in light. Over time, microbial activity and abiotic processes result in chemical and environmental gradients from top to bottom and surface to interior of the columns, resulting in diverse niches for microbial growth. Light serves as the energy source for primary producers and a structured microbial ecosystem develops in which all the necessary processes occur to maintain nutrient cycling. Winogradsky columns are frequently used in undergraduate microbiology courses to demonstrate or investigate microbial metabolic diversity [23–25] but have also been used for other applications including enrichment or isolation of novel bacteria [26,27], bioremediation [28], and generation of biohydrogen [29]. The Winogradsky column may also be a useful model microbial ecosystem to study environmental influences on microbial community structure and dynamics, as the complex community can be maintained or manipulated under carefully controlled laboratory conditions.

In a previous study, we used high throughput 16S rRNA gene sequencing to examine the microbial community of Winogradsky columns prepared with different sediment and supplemental cellulose sources [30]. The structure of the community was found to be strongly dependent on the sediment source and depth within the column, leading us to propose that the Winogradsky community is formed by a founder effect followed by diversification by depth. In the current study, we investigated the dynamics of community assembly in Winogradsky columns by measuring the changes in the microbial community over time. We show that the Winogradsky column microbial community quickly changes from that of the founding sediment and continues to change over time, ultimately leading to the development of a unique surface biofilm that is highly enriched in taxa that are initially rare. In addition, we propose that bacteriophages may play a role in the microbial dynamics of a Winogradsky column.

## Methods and Materials

### Site description

Permission to sample on Vassar College campus was given by Vassar College. Sediment was collected below approximately 6” of water from Sunset Lake, a pond on Vassar College campus (41°40'59.6"N 73°53'33.5"W) on July 15, 2013. The Casperkill Creek is dammed to form Sunset Lake. The creek begins in wetlands approximately 2 miles north of the lake and flows through a suburban area before reaching the college campus [31]. There are mixed trees near the lake but they do not shade the lake surface.

### Column and sample preparation

Sediment was sifted through a 0.25” soil sifter to remove stones and other large debris. Three samples of this sediment were collected and frozen to serve as the “pond” (time = 0 d) samples. Enriched sediment was prepared by adding 4.5 g dried leaf litter, 5 g CaSO_4_, and 5 g CaCO_2_ per 100 ml sediment. Acrylic columns (5.5 cm diameter, 18 cm height, Carolina Biologicals) were filled to a depth of 4 cm with enriched sediment, then filled to approximately 12 cm with unenriched sediment, forming the bottom and top layers respectively, to help establish a steeper sulfide gradient within the column. Visible air bubbles were removed using a fine-tipped spatula. The top of each column was covered with plastic wrap held by an elastic band. Fifteen columns were used for the time series, three for each time point. All columns were incubated in a Conviron E15 incubator at 25°C, 60% relative humidity, with 24 h/d illumination using Phillips F72T12/D/HO/Alto fluorescent bulbs, for 3, 9, 18, 39, or 60 d. One additional column was prepared and incubated for 60 d to analyze the surface and interior communities. Two additional columns were wrapped in aluminum foil to serve as the “dark” columns incubated without light. Foil-wrapped columns were incubated for 60 d in the same incubator. After incubation all columns were frozen at -20°C until used for DNA extraction.

Frozen columns were cut into two pieces at the interface between the top and bottom layer using a band saw. Each piece was then individually thawed and mixed to homogeneity before taking a sample. Thawed sediment was easily extruded from the acrylic by gently taping it on a beaker or pushing with a gloved hand. Any material left attached to the walls of the acrylic was scraped and added to the extruded sediment. Three replicate samples were taken from the top layer of a 39 d column and duplicate samples were taken from the top and bottom of an 18 d column. To obtain surface and interior samples, a column incubated for 60 d was partially thawed and removed from the acrylic. The surface layer, defined here as the exterior layer of the sediment adjacent to the acrylic, was approximately 1–3 mm thick and was carefully scraped and homogenized. The remaining sediment, lacking the surface layer, was then thawed and homogenized to form the “interior” samples. Triplicate surface and interior samples were taken.

### DNA extraction, amplification and sequencing

DNA extraction was performed on 40–100 mg of sediment using a MoBio PowerSoil DNA isolation kit (MoBio, CA) following the manufacturer’s directions. The V4 region of the 16S rRNA gene was amplified by PCR using a high-fidelity polymerase (Platinum Pfx polymerase, Life Technologies, Grand Island, NY) and barcoded 515F and 806R primers with Illumina flowcell adaptor sequences as previously described [32,33]. Each 25 μl reaction contained 2 μl extracted genomic DNA, Enhancer solution at a 2X final concentration, 0.8 μM of each primer, 1 mM MgSO_4_, 0.3 mM dNTPs, and PCR buffer at a final 1X concentration. PCR cycling was as follows: 94°C for 5 min, then 32 cycles of 94°C for 15 s, 50°C for 45 s, 68°C for 30 s. Sequencing was performed at Cofactor Genomics (St. Louis, MO) using Illumina’s MiSeq to generate paired-end reads. Sequences were deposited in NCBI (BioProject ID: PRJNA272390).

### Quality filtering and OTU picking

All processing and data analysis was performed using the Quantitative Insights Into Microbial Ecology software package (QIIME, v1.6.0 or v1.8.0) [34]. Low quality reads were removed at the default Q25 setting and samples were demultiplexed. Open reference operational taxonomic unit (OTU) picking was performed by using UCLUST with clustering at 97% sequence identity. Representative sequences were chosen for each OTU, and taxonomic identities were assigned using the RDP Classifier [35] retrained with the greengenes taxonomy in QIIME using default settings [36]. Chimeric sequences were removed using ChimeraSlayer. Following all quality filtering steps, 6.26 million sequences remained of the original 8.75 million. Four samples (one each of top layer dark, bottom layer dark, 39 d bottom layer, 18 d bottom layer) were excluded from further analysis due to low sequence counts (less than 1700), resulting in a final set of 52 samples. Additional filtering for sequence errors was performed by removing OTUs containing fewer than 50 total sequences.

### Diversity analysis

Alpha diversity was calculated using the Shannon index, OTU richness, Berger-Parker Dominance index, and PD whole tree index in QIIME. Rarefaction curves were generated by repeated (10 times) subsampling of 50 to 15000 sequences, with 11 steps from minimum to maximum sampling depth. Nonparametric two-sample t-tests were used to test for significant differences in alpha diversity. The default number of Monte Carlo permutations (999) were used to calculate p-values in the nonparametric t-tests, and the Bonferroni correction was used with α = 0.05.

Phylogenetic beta diversity was calculated using both unweighted and weighted UNIFRAC [37,38] at a depth of 15000 sequences per sample. Principal coordinate (PCoA) plots were generated from the distance matrices. Statistical analysis of UNIFRAC distances was performed using JMP software. Average distances were calculated between replicate sample pairs, then analyzed by ANOVA followed by Tukeys-HSD test. Alpha was set at p = 0.05. Biplots were generated in QIIME using the script make_emperor.py.

Heatmaps were generated using the heatmaps.2 function from the gplots R package in R [39]. Genera with a maximum abundance of less than 1% in all samples were removed. Abundances were scaled and centered using the scale function.

### Conditionally rare taxa (CRT)

CRT, taxa that show rare-to-prevalent dynamics, were analyzed using CRT analysis [40]. To identify CRT in the time series, samples were rarefied to 17000 sequences, slightly less than the sample with the minimum number of OTUs, and then summarized at the taxonomic rank of genus using summarize_taxa.py in QIIME; thus CRTs in this case are conditionally rare genera. Abundances in replicate samples were averaged, and then CRT analysis was performed using a coefficient of bimodality of 0.7 (rather than the default of 0.9, to allow detection of taxa that gradually increase or decrease in abundance) and an abundance threshold of 0.5%.

CRT analysis was also applied to spatially distributed samples of the core and surface communities, where the condition is location, rather than time. Samples were rarefied to 40000 sequences, slightly less than the sample with the minimum number of OTUs. Subsequent analysis of spatially distributed CRT was performed as described above for the time series CRT.

### Time-lapse photography of Winogradsky panel

In addition to the traditional cylindrical Winogradsky column, we constructed a thin, panel version of this ecosystem model. The panel format was chosen to reduce surface glare and to have a flat plane of focus for photography. The Winogradsky panel was made using 12” x 24” x 0.5” acrylic sheets separated by 0.25” thick 1” wide acrylic trim. An opening was left at the top of the panel. Sediment was collected and prepared as for the cylindrical columns, except with additional sifting using a 1/8” soil sifter and addition of excess pond water to decrease viscosity to more easily pour the sediment. Enriched sediment was poured to a depth of 4 cm, and the panel was subsequently filled with unenriched sediment. The panel was kept at room temperature and illuminated using 24W/6400k T5 fluorescent lamps.

. A time-lapse camera (Brinno TLC100) was used to capture 1 image every 24 h. Each image contained a timestamp. The movement of plaque edges was tracked and analyzed using Logger Pro v3.8.6.1 (Vernier Software & Technology). A ruler was attached to the panel to serve as calibration for distances seen in the images. Plaque expansion was analyzed in several directions using Logger Pro’s position versus time video function. Coordinate axes were plotted directly onto the video with the origin positioned at the location where bacterial clearance was first seen. Coordinate dots were positioned at the outer periphery of the plaque, along the rotatable axis. One coordinate dot was placed for each day the plaque spread. A linear fit of the plaque edge position coordinate over time was measured to determine average speed of plaque expansion in each direction.

## Results and Discussion

### Time-lapse photography of panel and description of columns

Time-lapse photography of a Winogradsky panel was used to document visible changes over time (S1 Movie). Slight changes were already apparent after 1 d, followed quickly by more pronounced changes along the interface between the enriched and unenriched sediments. Formation of iron oxide and growth of green, brown, and other pigmented colonies were apparent throughout. Darkening of the enriched sediment due to hydrogen sulfide production occurred within the first week. Changes were rapid early on, and slowed down but continued for the duration of the experiment, a total of 248 d. We noted a period beginning at 30 d in which many colonies disappeared, and describe this pattern in more detail later in the paper.

**Fig 1:**
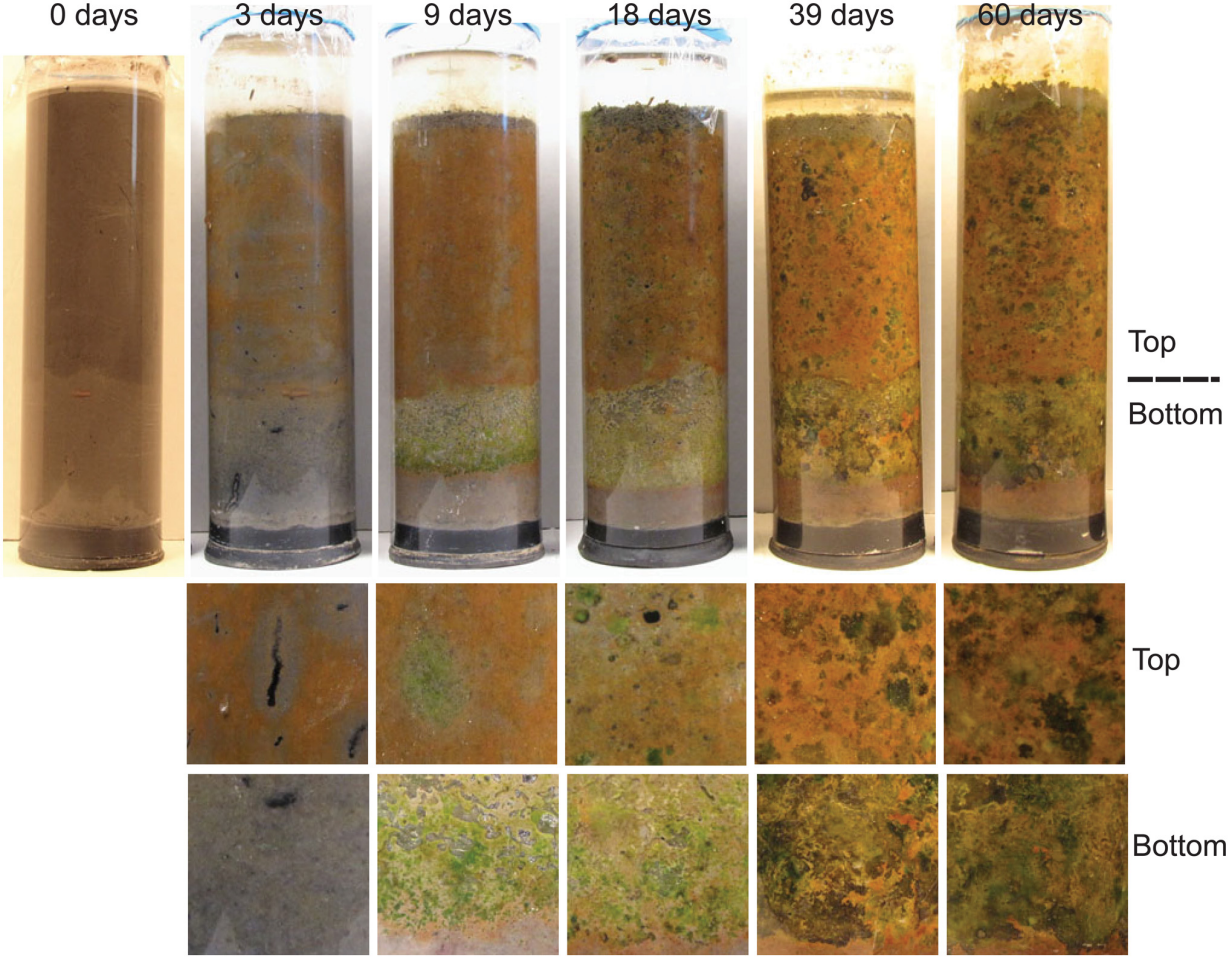
Development of Winogradsky columns over time. Columns were prepared with pond sediment and incubated with continuous illumination for the indicated time. The demarcation between the enriched (bottom) and unenriched (top) sediment layers is visible and indicated at the right. Bottom images show details of representative regions of the top and bottom layers.

We also prepared Winogradsky columns to sample the microbial community over time. Visible changes were apparent in the columns by 3 d as superficial regions of orange color developed in the top layer, most likely iron oxides (Fig 1). Progressing to 60 d, both top (unenriched) and bottom (sulfate enriched) layers increased in complexity and diversity of pigmented colonies, and a clear demarcation was present between the bottom sediment and top sediment. Replicate columns were very similar to each other. We note that these columns are visibly very different from those used in a previous study, which used different sediment sources [30].

### Sequencing and taxonomic assignment

DNA was extracted from samples collected from pond sediment (time = 0 d) and columns incubated for 3, 9, 18, 39 and 60 d. 16S rRNA genes were amplified by PCR and sequenced via Illumina sequencing. After quality and chimera filtering, there were 5.75 million reads of average read length 251nt in 52 samples with at least 17000 reads each. Four samples that had less than 1700 sequences were excluded from further analysis.

Reads were assigned to OTUs using UCLUST and then classified with the greengenes database as implemented in the QIIME pipeline. A total of 9517 OTUs were detected and classified into 64 phyla and 822 genera. 11 of the phyla constitute 90% of the total community, indicating that most phyla are quite rare. To characterize the most abundant members of the community, we defined abundant genera as those that make up at least 1% of the community of at least one sample. The top and bottom layers contained 41 and 40 abundant genera, respectively, from 11 phyla. 9 genera were abundant only in the top layer, 8 were abundant only in the bottom layer, and 32 were abundant in both.

### Winogradsky column community changes over time

**Fig 2:**
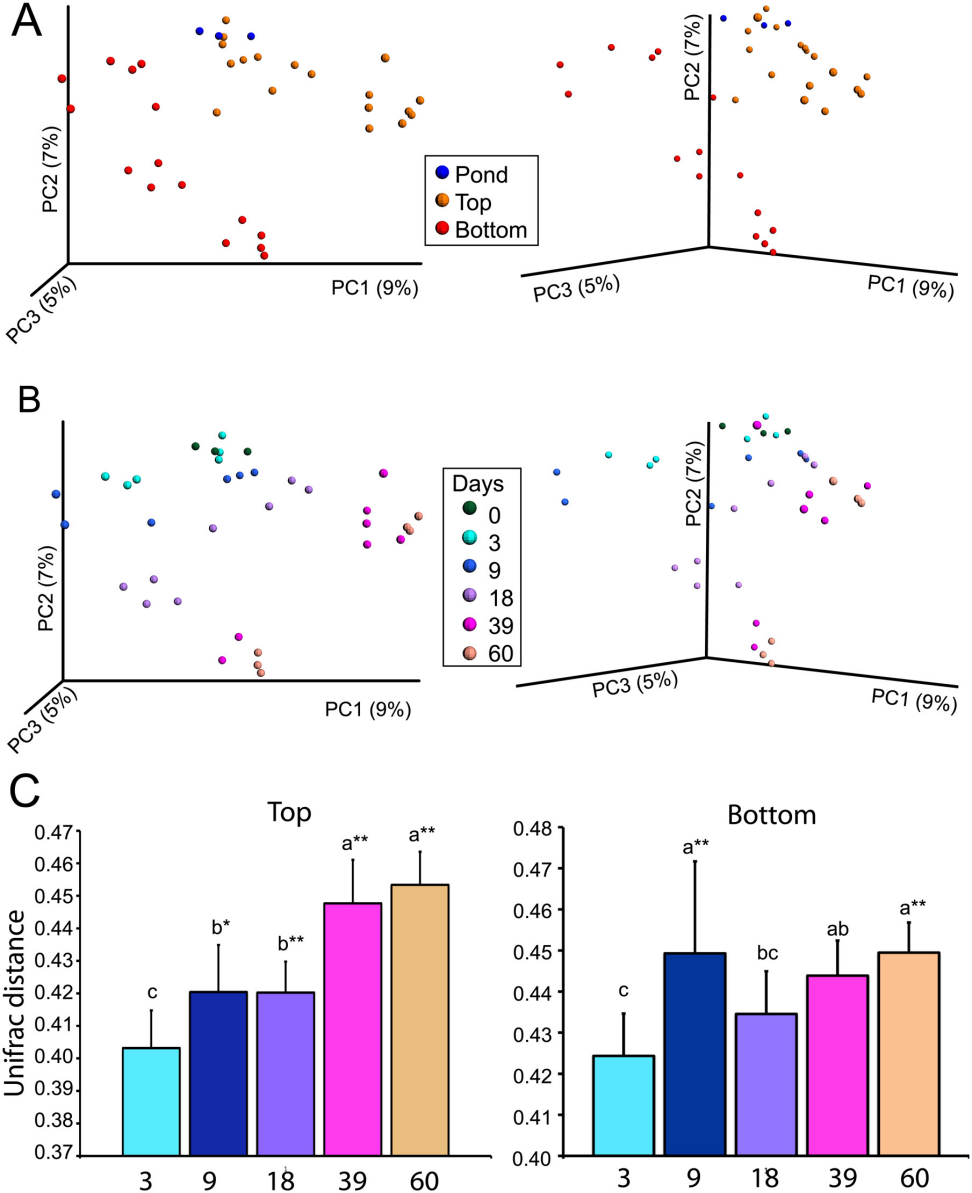
Phylogenetic diversity in developing Winogradsky columns. Unweighted UNIFRAC and PCoA were used to evaluate the phylogenetic similarity between samples. Samples are colored by A) location or B) time of incubation. Axes indicate percent of variation explained by the principle coordinate (PC). The figures on the right are a rotated view of the left figure to show separation along PC3. C) Average UNIFRAC distance between 0 d (pond) and time of incubation shown, in top (left) and bottom (right) column layer samples. Top: groups joined by the same letter (a, b, or c) are not significantly different (ANOVA, Tukeys-HSD, df = 53). ** p<0.01 compared to 3 d, * p<0.05 compared to 3 d. Bottom: groups joined by the same letter (a, b, or c) are not significantly different (ANOVA, Tukeys-HSD, df = 44). a** p<0.01 compared to 3d, all other significant differences have p<0.05.

To evaluate the changes in the community over time we used the UNIFRAC metric [38] and principal coordinate analysis (PCoA), which measures between-sample phylogenetic diversity (beta diversity). Over time, the structure of the Winogradsky column community diverged from the founding pond sediment community as shown by separation of samples along the first principle coordinate axis (PC1) (Fig 2 and S1 Fig). Enrichment also affected the rate and extent of change. After 3 d the bottom (sulfate enriched) layer diverged slightly, while the top layer remained similar to the pond sediment until 9 d of incubation. Later time points were further separated from 0 d. Top and bottom layer samples clustered separately along the second principal coordinate axis (PC2).

We also compared the average UNIFRAC distance between the pond (t = 0) and each time point to evaluate the change in community over time, and found that, over time, the community diverges significantly from the founding population (Fig 2C). In the top layer, the community at 9 d and 18 d was more phylogenetically distant from the pond community than the community at 3 d, and the community of 39 d and 60 d columns was more phylogenetically distant from the pond community than all preceding timepoints (p<0.01). In the bottom layer, a similar pattern was observed. These differences indicate that the community changes gradually over time, becoming increasingly different from the pond community. The lack of a difference between the last two timepoints in both the top and bottom layers suggest that changes in the column were slowing down and that the columns may have reached a stable community, however additional timepoints beyond 60 d would be necessary to confirm this.

While the between-sample phylogenetic diversity changed with time and enrichment, the within-sample (alpha) diversity of Winogradsky column samples and pond sediment samples did not differ (non-parametric t-tests, p>0.05). Pond and column samples were highly diverse; the Shannon index of all samples was greater than 10 at a rarefaction depth of 15000 sequences. The species richness, Berger-Parker dominance index, and phylogenetic diversity (PD whole tree) index, also did not differ by time or location (non-parametric t-test, p>0.05). Analysis of top and bottom layers separately also did not reveal differences in alpha diversity over time. This indicates that the composition but not the overall diversity of the Winogradsky column community changed over time.

**Fig 3:**
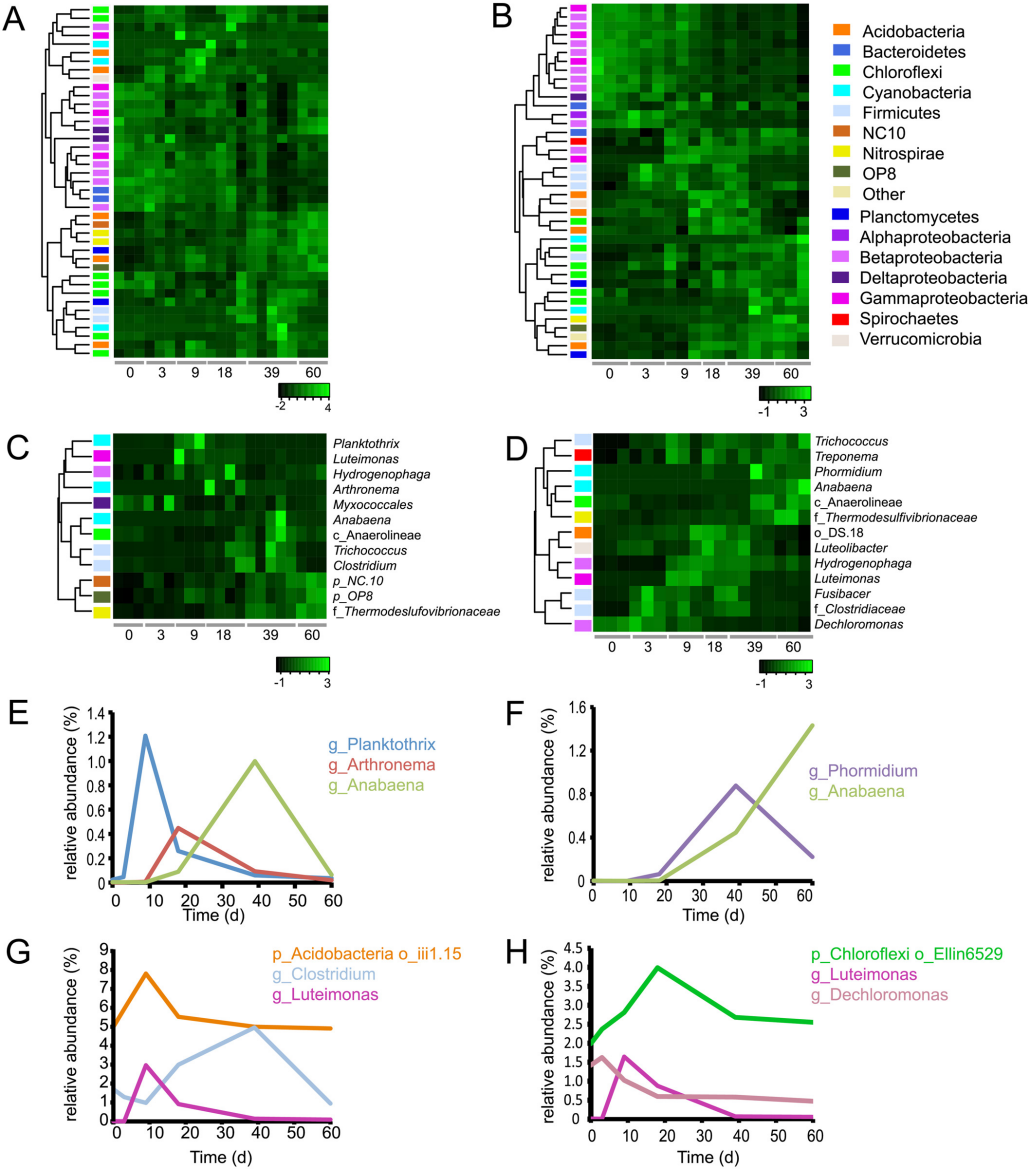
Time dependent changes in relative abundance of genera in Winogradsky columns. OTUs were taxonomically assigned and filtered to include only genera that represent at least 1% of the community of at least one sample. Relative abundances were normalized, and heatmaps were generated to show Z-scores. Rows were clustered according to abundance pattern over time. Abundant genera in the A) top layer and B) bottom layer are shown. Each row represents a unique genus and the colored bar on the left represents the phylum to which it belongs. C and D) Genera that were enriched at least 5-fold in any sample of the top (C) and bottom (D) of the column. Selected taxa that showed rare-to-prevalent dynamics as determined using CRT analysis: E) Cyanobacteria, top layer; F) Cyanobacteria, bottom layer; G) other taxa, top layer; H) other taxa, bottom layer. For clarity, the prefixes p_,c_,o_, f_, and g_ are used to denote phylum, class, order, family and genus, respectively.

To describe the changing structure of the Winogradsky columns, OTUs were assigned to taxonomies using QIIME. Most genera of the phylum Proteobacteria decreased in relative abundance over time, while genera of Cyanobacteria, Chloroflexi, Nitrospirae, and Planctomycetes increased in abundance over time, reaching maxima at 39 or 60 d (Fig 3). Proteobacteria were the most abundant phylum comprising 40.3% of the pond sediment (time = 0 d) community, but declined in relative abundance to 26.5% and 34.5% of the bottom and top layer of 60 d columns respectively.

Since the Winogradsky column is considered an enrichment culture, we were interested in identifying taxa that showed the most pronounced changes over time. We approached this in two ways. First, we identified taxa that showed at least 5-fold difference in relative abundance between any two samples and represented at least 1% of the population. Twelve and 13 genera met these criteria in the top and bottom layers, respectively, 6 of which are present in both (Fig 3C and 3D). All were taxa that were enriched in the columns compared to the pond sediment. Among them were several heterotrophs, including *Fusibacter*, *Dechloromonas*, *Trichococcus*, and *Luteimonas*, that are likely to function in the carbon, sulfur, or nitrogen cycles through their diverse metabolic activities [41–45]. The Spirochete *Treponema* was also enriched by 9 d. Some species of *Treponema* form close associations with cellulolytic bacteria [46], so its enrichment here is likely coupled to ongoing degradation of cellulose.

Phototrophs were also enriched. *Rhodobacteria*, one of the “purple bacteria” uses H_2_S as an electron donor in anaerobic phototrophy. Two genera of Cyanobacteria (*Anabaena* and *Phormidium*) were enriched at later time points. The production of oxygen through photosynthesis by abundant Cyanobacteria at these later time points may be responsible for the decline in relative abundance of some of the anaerobes such as *Clostridiaceae*. Certain Chloroflexi, the “green non-sulfur bacteria,” are phototrophic, however, the abundant Chloroflexi genera in these columns are members of the non-phototrophic class Anaerolinea.

A few genera are uniquely enriched in the top layer (Fig 3C). Among these are two genera belonging to candidate phyla NC10 and OP8, which lack representatives grown in pure culture, and that increase in abundance at 39 and 60 d. The candidate phylum OP8 is found in diverse habitats and environmental conditions, but typically in very low relative abundance. A meta-analysis of high-throughput sequencing studies found an average OP8 relative abundance of 0.146% in non-marine aquatic habitats [47], and these Winogradsky columns have 1.0% OP8 at 60 d. Candidate phylum NC10 includes members grown in enrichment culture that are capable of anaerobic methane oxidation coupled to denitrification [48,49].

The second approach to characterizing the taxa that change in prevalence was to identify conditionally rare taxa (CRT) [40]. CRT are taxa that show a rare-to-prevalent trajectory in a time series or under different environmental conditions, resulting in a bimodal distribution. There were 26 CRT in the top layer, and 40 CRT in the bottom layer, some of which are shown in Fig 3E–3H. This rare-to-prevalent pattern is particularly notable in the Cyanobacteria, as the drop in one Cyanobacteria genus coincides with the increase in another. In the bottom layer, *Phormidium* grows rapidly between 18 d and 39 d, and then drops as *Anabaena* increases to a maximum at 60 d (Fig 3F). In the top layer, a succession of *Planctothrix*, *Arthronema*, and *Anabaena* takes place from 9 d to 39 d (Fig 3E). This pattern suggests that as one Cyanobacteria taxon declines in abundance, another rises to occupy the available niche.

### A unique surface community develops

**Fig 4:**
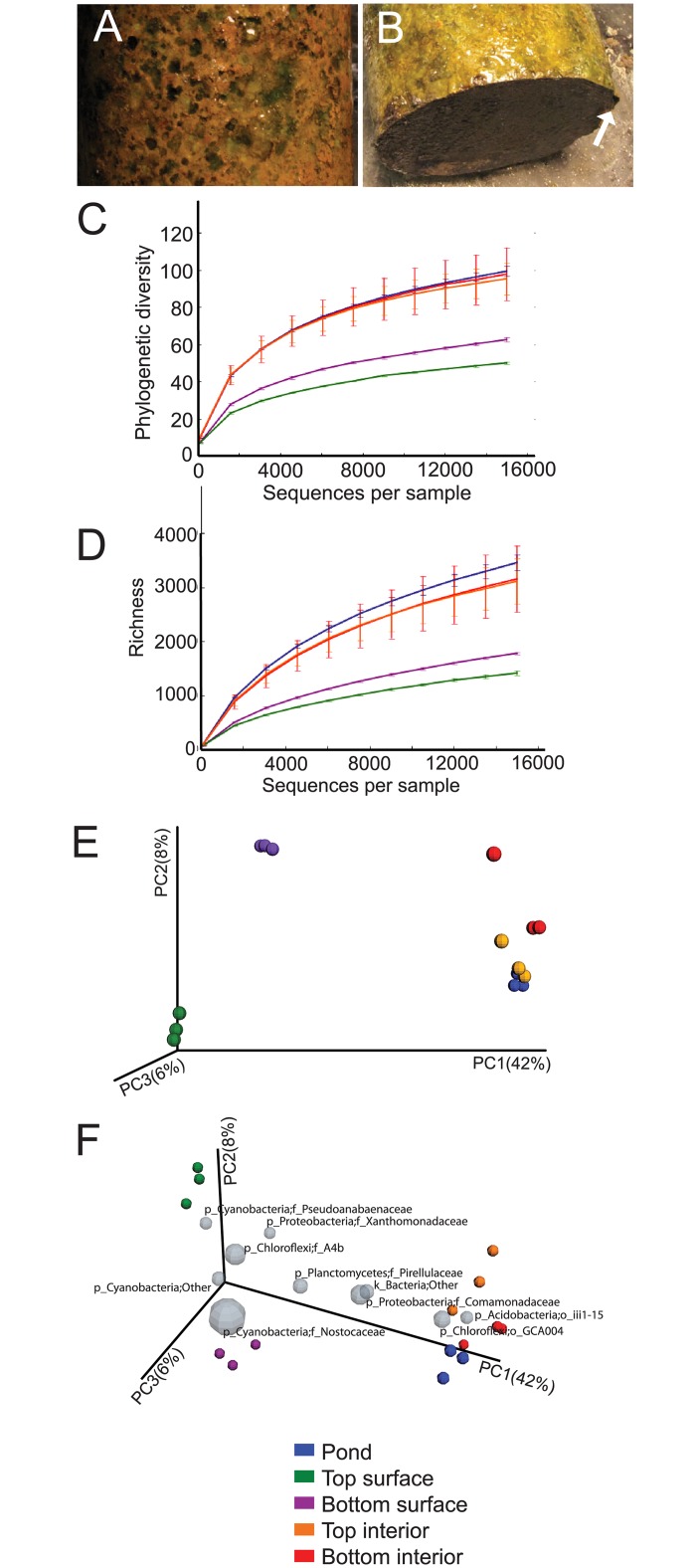
Surface community of Winogradsky columns after incubation for 60 days. A) Top and B) bottom slices of a partially thawed column showing the surface layer. In B, the distinction between the thin surface layer and the interior is apparent at the edge of the slice, shown by an arrow. Surface and interior sequences were rarefied to depths of 50 to 15000 sequences and alpha diversity metrics C) PD whole tree (phylogenetic diversity) and D) species richness were used to assess diversity. E) Unweighted UNIFRAC and PCoA were used to assess between-sample diversity of samples rarefied to a depth of 15000 sequences per sample. F) A biplot showing the 10 most abundant family-level taxa. View is rotated from perspective in E. Grey spheres represent taxa; the size of the sphere is proportional to the average relative abundance. The coordinates of a given taxon are plotted as the weighted average of the coordinates of all samples and the weights are the relative abundances. Percent variation explained by each PC axis is shown.

**Fig 5:**
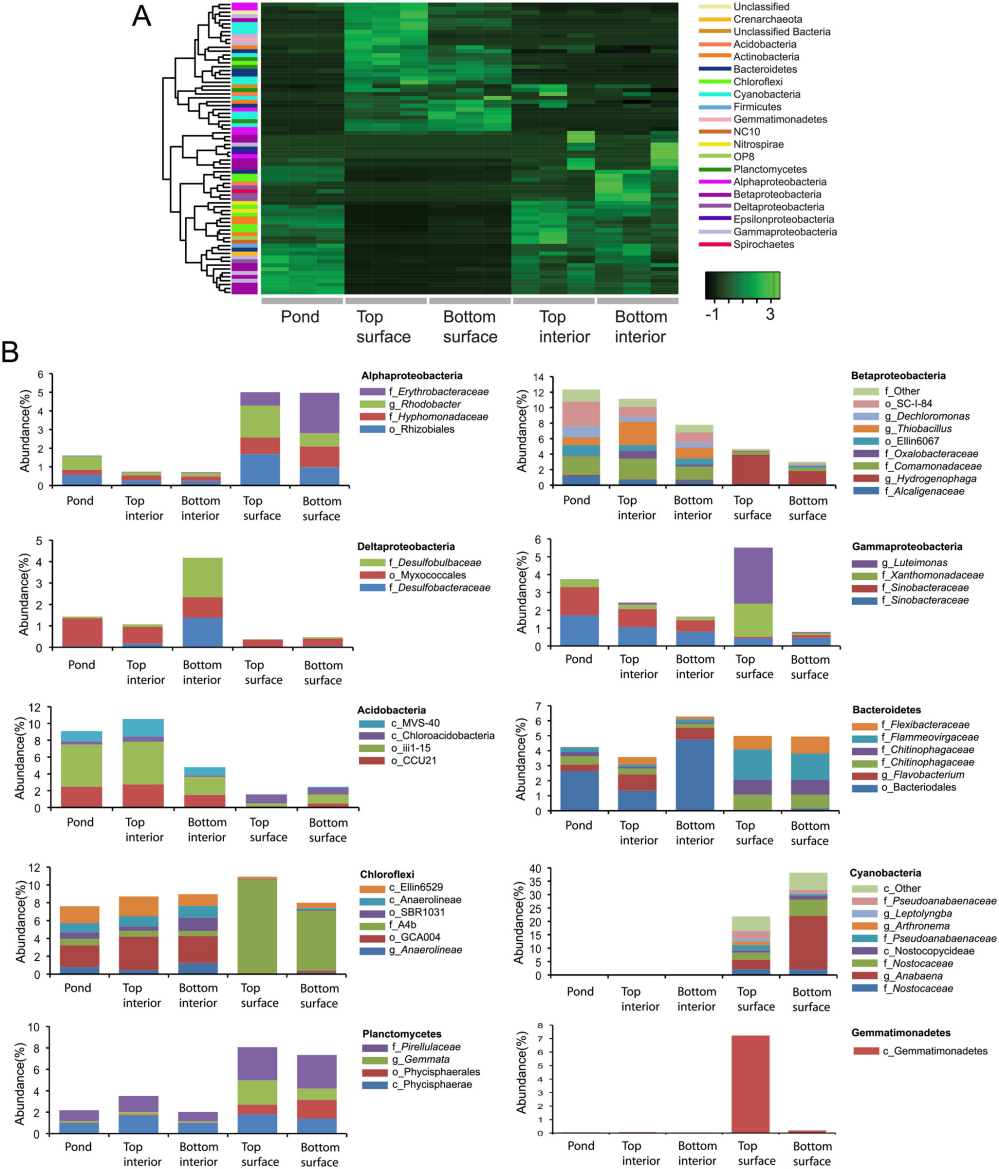
Structure of the surface, interior and pond communities. OTUs were taxonomically assigned and filtered to include only genera that represent at least 1% of the community of at least one sample. A) Relative abundances were normalized, and heatmaps were generated to show Z-scores. Rows were clustered according to abundance pattern. Each row represents a unique genus and the colored bar on the left represents the phylum to which it belongs. B) Relative abundance of genera in the pond, surface and interior, grouped by phylum. Each colored portion of the stacked bars represents a unique genus, however the taxonomic name given in the keys is for the lowest named taxonomic rank to which the genus belongs. For clarity, the prefixes c_,o_, f_, and g_ are used to denote class, order, family and genus, respectively. Note that only the most abundant genera are shown, so the sum of the genera for a particular phylum does not necessarily represent the total abundance for that phylum.

When preparing the columns for sample extraction we noted that a surface biofilm community developed between the sediment and acrylic that could be peeled off of a partially thawed column (Fig 4A and 4B). We analyzed the surface and interior communities of a 60 d column separately, and found that the surface community was less diverse than the interior and was enriched in taxa that are distinct from those of the interior community and pond sediment (Fig 4C–4E and S1 Fig). A biplot was generated to show the 10 most abundant family-level taxa (Fig 4F). The coordinates of a taxon on the plot are determined by the weighted average of the coordinates of all samples, where the weight is the relative abundance, and therefore shows the taxonomic drivers that differentiate samples. Most notably, several Cyanobacteria and a Planctomycetes cluster near the surface samples, while Acidobacteria cluster with the interior and pond samples. Different families of Chloroflexi and Proteobacteria also differentiate the surface from the interior and pond samples. Further comparison of the most abundant members of the surface and interior communities showed little similarity (Fig 5A). The interior of the column was similar to the pond, indicating that the most significant changes in the Winogradsky column occurred in the surface layer.

The surface community was comprised of metabolically and structurally diverse bacteria. Photosynthetic Cyanobacteria made up slightly more than 40% of the bottom surface and 25% of the top surface (Fig 5B). Alphaproteobacteria, including *Erythrobacteraceae* and *Rhodobacter* were present and *Hydrogenophaga* dominated the Betaproteobacteria. *Erythrobacter* is an anoxygenic phototroph containing bacteriochlorophyll a, giving it a red-orange color [50]. *Rhodobacter* species, part of the traditional group of purple bacteria, are metabolically diverse; they are capable of phototrophy, aerobic and anaerobic respiration, fermentation and nitrogen fixation [51]. Planctomycetes, which lack peptidoglycan cell walls, made up almost 12% of the surface community. The relative abundance of Bacteroidetes is similar in the interior and surface, but different genera are present. Chloroflexi made up approximately 11% of the top and bottom surface layers, a similar proportion to interior and pond communities, but less diverse. Chloroflexi in the surface layer are dominated by a single member of the family A4b in the class Anaerolinea. Cultured representatives of the Anaerolinea are slow growing anaerobic chemolitho- or organoheterotrophs [52]. It is interesting to find an anaerobe in high abundance and in close proximity to Cyanobacteria. However, Cyanobacteria are capable of anaerobic respiration and anoxygenic photosynthesis using H_2_S as an electron donor [53]. Enrichment of the lower layer of sediment with calcium sulfate promotes H_2_S production by sulfate reducers and may encourage the use of anoxygenic photosynthesis. Further, steep chemical gradients have been observed over millimeter distances in Cyanobacterial mats [53], suggesting that the Winogradsky column surface biofilm may also have microenvironments that allow interaction among organisms with diverse metabolic requirements.

**Fig 6:**
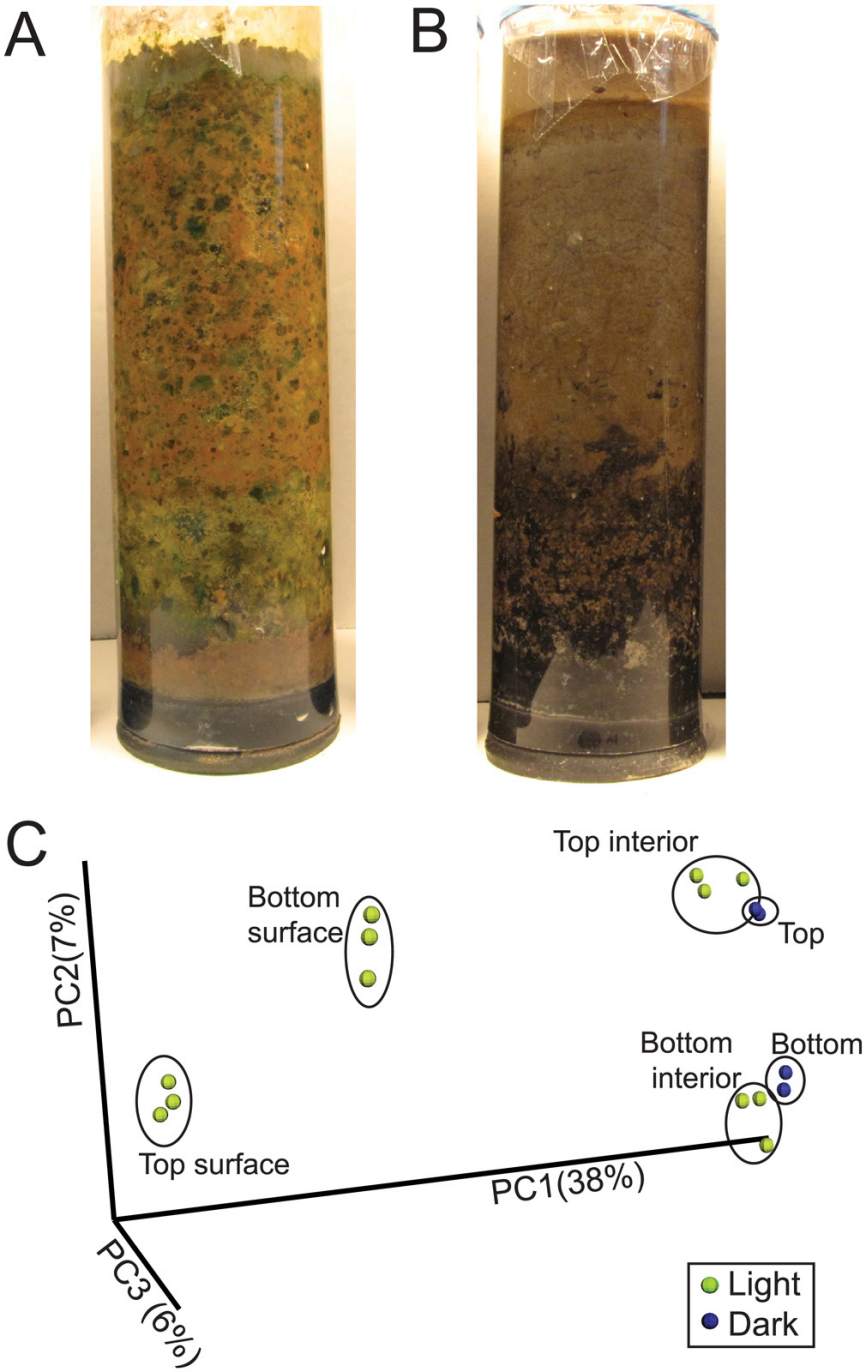
Comparison of Winogradsky columns incubated with or without light. Column incubated for 60 d A) with light or B) without light. C) Unweighted UNIFRAC and PCoA were used to assess between-sample diversity of samples rarefied to a depth of 15000 sequences per sample. Percent variation explained by each PC axis is shown.

Not all members of the surface community are phototrophs, but development of the surface community was dependent on light (Fig 6 and S1 Fig). Columns incubated wrapped in foil to exclude light did not develop the visible surface community and the structure of the community was similar to those of the interior and pond. The similarity of the dark column community to the interior of light-incubated columns suggests that the activity of the surface community may have had relatively little impact on the interior.

It was in the surface layer that enrichment in the Winogradsky column was most apparent. Using CRT analysis, we identified 38 taxa that were rare in the pond but prevalent in the top surface. In the pond, only 5 were present at greater than 0.5%, and none greater than 1%, in total representing 7.4% of the pond population. In the surface layer, however, these same taxa made up 72% of the community. In the bottom, 37 CRT were identified that made up 67% of the surface community. The genera that showed at least 5-fold enrichment in the surface are shown in Table 1. The most highly enriched were the Cyanobacteria, showing almost 1000–10000 fold enrichment. A member of the phylum Gemmatimonadetes comprised less than 0.05% of the pond sediment community and more than 7% of the top surface community. Few members of this phylum have been cultured, and are typically found in greatest abundance in dry, arid soils [54], a sharp contrast to the very wet Winogradsky sediment. These findings supports the hypothesis that rare taxa can serve as microbial seed banks [55,56] and can grow to abundance under appropriate conditions. Further, given the metabolic diversity of taxa in the surface biofilm, the structure and interactions among individuals in the surface biofilm must create local conditions that are highly favorable for specific taxa.

**Table 1:** Taxa enriched at least 5-fold in 60 day column surfaces compared to pond sediment.

Taxonomy		Abundance (%)			Fold increase relative to pond	
Phylum	Name	Pond	Bottom surface	Top surface	Top surface	Bottom Surface
Bacteriodetes	f_Flammeovirgaceae	0.30	1.78	2.01	**7**	6
Chloroflexi	c_Anaerolineae;f_A4b	0.71	6.72	10.52	**15**	9
Cyanobacteria	f_Nostocaceae	0.01	1.97	2.17	**246**	223
Cyanobacteria	f_Pseudanabaenaceae	1.52 x 10^−3^	0.62	1.93	**1267**	408
Cyanobacteria	g_Arthronema	3.04 x 10^−4^	0.06	1.36	**4457**	209
Cyanobacteria	g_Leptolyngbya	6.08 x 10^−4^	0.24	1.02	**1682**	391
Cyanobacteria	f_Pseudanabaenaceae;Other	3.04 x 10^−4^	1.15	3.03	**9979**	3777
Gemmatimonadetes	c_Gemmatimonadetes	0.05	0.20	7.23	**156**	4
Planctomycetes	g_Gemmata	0.13	1.06	2.27	**17**	8
Proteobacteria	g_Hydrogenophaga	0.12	1.70	3.81	**31**	14
Proteobacteria	g_Luteimonas	0.02	0.07	3.14	**199**	5
Cyanobacteria	g_Anabaena	2.74 x 10^−3^	20.05	3.46	1266	**7328**
Cyanobacteria	f_Nostocaceae	0.01	6.11	2.64	394	**914**
Cyanobacteria	f_Nostocophycideae	3.04 x 10^−4^	1.47	0.92	3034	**4826**
Cyanobacteria	p_Cyanobacteria;Other	9.12 x 10^−4^	6.53	5.33	5838	**7161**
Planctomycetes	o_Phycisphaerales	0.05	1.76	0.92	17	**32**
Proteobacteria	f_Erythrobacteraceae	0.03	2.17	0.72	28	**84**

The Winogradsky columns used in this study were visibly different, and had different communities, than those used in our previous study [30]. The primary difference between these studies was the sediment source used: Sunset Lake (NY) in this study, and Eph’s and Buxton Ponds (MA) previously. Our previous work demonstrated that sediment, source plays a major role in determining the Winogradsky community, and the current study supports this finding. It is unknown at this time, however, what specific differences in properties or nutrients among the sediments are responsible for the differences. We also used a different sampling approach in the current study. In our previous study, samples were collected by drilling into the column, while in the current study, samples were collected by cutting frozen columns. This approach revealed similar communities from samples from replicate columns and replicate samples from the same column, as seen in PCoA plots (Figs 2 and 4), suggesting the technique yields reproducible results. In a pilot experiment, a Winogradsky column made using Sunset Lake sediment was sampled by drilling and 16S rRNA sequencing revealed a community similar to that seen in the current Sunset Lake columns (data not shown), indicating that the sediment source, not the sampling technique, can explain the difference from the Buxton Pond and Eph’s Pond Winogradsky column communities, This approach has several advantages. By drilling, the surface layer may be destroyed, resulting in collection of primarily interior material, and it is impossible to separate surface and interior layers. Further, possible microheterogeneity between sites in a column is likely to have a more significant effect on sample reproducibility when collected by drilling than collecting whole layers.

### Possible drivers of community dynamics in Winogradsky columns

The specific mechanisms behind changes in abundance of individual members of the microbial community are unknown. However, community assembly is likely to involve a combination of deterministic niche effects (competition and habitat selection, for example) and stochastic processes (such as growth rates, death rates, or dispersion) [3,57,58]. Initial changes in the Winogradsky community are likely the result of increased exposure to light due to illuminated incubation and disturbance of the sediment caused by collection and preparation. This may alter local nutrient availability, interactions among cells, and introduce oxygen or other potential stressors. Although incubation conditions are maintained constant, and new organisms are not introduced, the environment within the column is expected to change as nutrients are consumed and metabolic products accumulate. The changing environment may result in less favorable conditions for initially highly active organisms and allow slower growing organisms to increase in abundance.

Bacteriophages have also been proposed to exert control over community diversity and host abundance in several ecosystems [18,19,21] in a predator-prey-like dynamic called “kill the winner” [59]. In this model, active and abundant bacteria are most susceptible to bacteriophage predation, resulting in a subsequent decrease in abundance. One possible explanation for the changes in dominant Cyanobacteria over time (Fig 3) could be sweeps of phages reducing the abundance of their host species, allowing another to grow. Future studies will need to address the possible roles of phage predation and environmental niche changes in decline and replacement of specific taxa,

In support of a role for bacteriophages in the dynamics of the microbial community of Winogradsky columns, close observation of the time-lapse video revealed areas of microbial colonization that were cleared from a central point outward (S2–S4 Movies). Numerous such zones were apparent during the period starting at 30 days and lasting approximately 2 months, resulting in a large reduction of surface-visible green-pigmented colonies, likely Cyanobacteria. The pattern is suggestive of bacteriophage plaque formation, and it is strikingly similar to time-lapse video microscopy of Vaccinia virus plaques in cell culture [60]. This period was preceded by rapid growth and a brief water leak from the panel, which introduced air into upper areas of the sediment.

**Fig 7:**
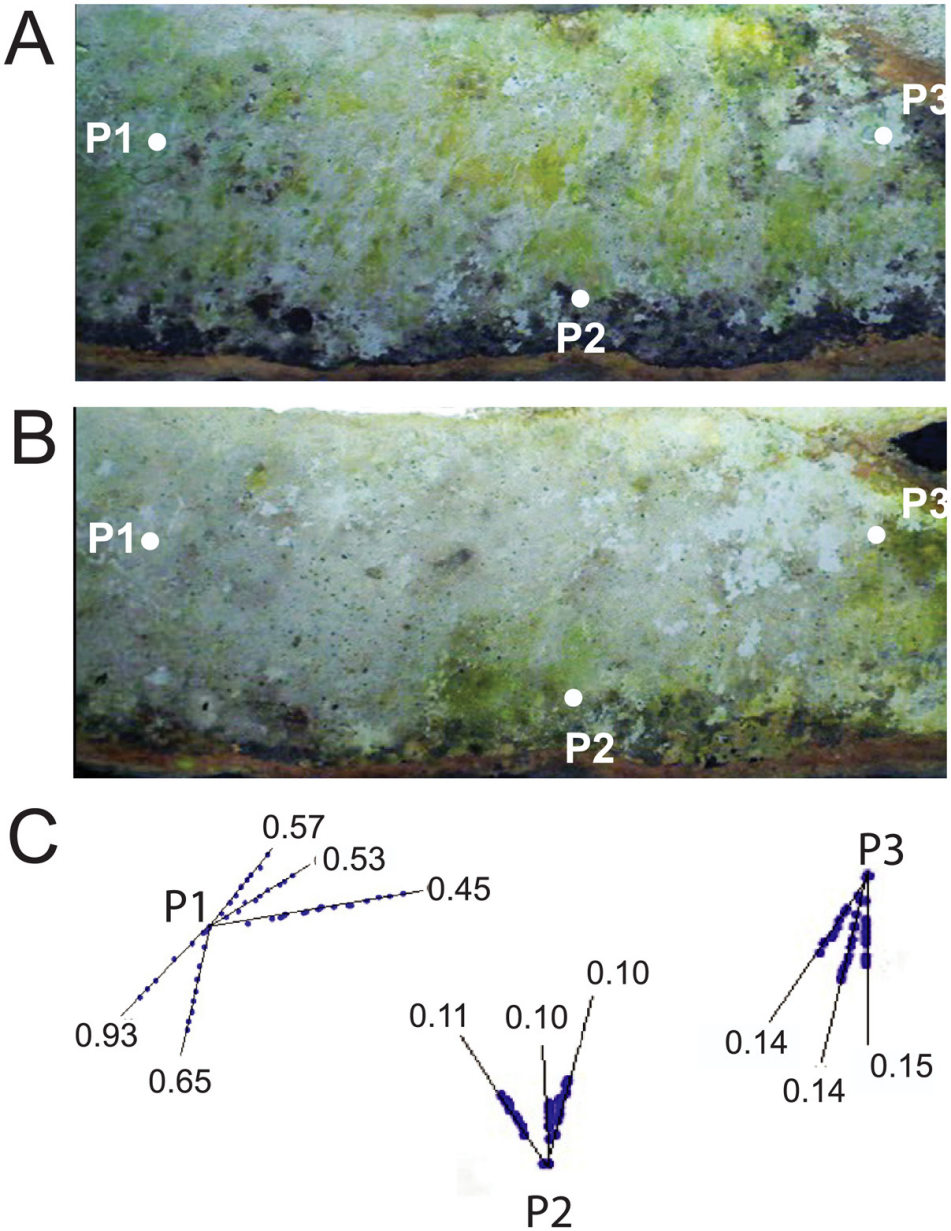
Zones of colony death in a plaque-like pattern. A) Still image from time-lapse video of a Winogradsky panel. B) The same area 53 d later. P1, P2, P3 indicate the points of origin of plaque-like zones tracked using LoggerPro software. C) The edges of plaques P1, P2, and P3 were tracked in sequential images using LoggerPro. Blue dots indicate the edge of the plaque-like zone in 1 d intervals along different vectors (black lines). Values show average distance traveled by the edge of the plaque-like zone along a given vector (cm/d). Edges were tracked until it was not possible to accurately mark its position, although the zones continued to grow beyond these points. Vectors of different zone origins are not shown to scale.

Through video analysis, we tracked the progression of the outer periphery of selected plaque-like zones of clearance. We found that they advanced at rates from 0.1 cm/d to 0.9 cm/d (Fig 7). Resolution of the images was insufficient to accurately track all edges. Future studies will be necessary to confirm the identity of these zones as bacteriophage plaques and demonstrate a functional role for bacteriophages in the shifting community structure of Winogradsky columns. Bacteriophages have been shown to be active and important in other ecosystems [18,19,61] and it would not be surprising to find that here as well.

## Conclusions

The Winogradsky column is a unique microbial ecosystem that has several advantages for use as a model system to study microbial and viral dynamics, interactions, and diversity. Once prepared, it is a self-sustaining, enclosed ecosystem dependent only on input of light as an exogenous energy source allowing for both short-term and long-term studies. We have found that Winogradsky columns prepared from the same sediment source form reproducible communities, which can be maintained and manipulated under controlled conditions. Formation of gradients by time and space (top to bottom and surface to interior) provide a unique opportunity to evaluate the development, alteration, and response of the microbial community to environmental variables. While not a simulation of a natural pond environment, which only receives light from above, the Winogradsky column may prove a useful model system to study important questions in microbial community ecology. In this study we used a 16S rRNA gene survey to characterize the microbial community dynamics during Winogradsky column development. Over a period of 60 days, the community changed from the founding population and formed a unique biofilm on the light exposed surface. The surface community was highly enriched in rare taxa indicating that rare taxa can become abundant under appropriate environmental conditions. The dynamics of certain taxa, most notably the Cyanobacteria, show a bloom-and-decline pattern, which is consistent with bacteriophage predation as predicted in the kill-the-winner hypothesis. Time-lapse photography also supported the possibility of bacteriophage activity in the Winogradsky community suggesting it may therefore be a useful model system to investigate both microbial and viral ecology.

## Supporting Information

S1 Fig Phylogenetic diversity in Winogradsky columns. 2D PCoA plots of unweighted UNIFRAC analysis showing first three principle component axes. A,B,C) Samples collected at indicated timepoints, colored by days of incubation. D,E,F) Samples collected at indicated timepoints, colored by location of sampling. G,H,I) Samples collected from surface or interior sections of Winogradsky columns incubated 60 days. J,K,L) Samples collected from columns incubated with or without light for 60 days.
(PDF)

S1 Movie Time-lapse series of Winogradsky panel. Images of a 12” x 24” panel were taken every 24 h with a time-lapse camera for a total of 248 days. Note that a 3-day gap is present (days 46–48 inclusive, with the dates 2012/11/02–2012/11/04). On day 28, the panel leaked water but not sediment, and C-clamps were used to stop the leak (visible beginning on frame 29 (2012/09/15)). The actual dates indicated at the bottom of each frame are incorrect, but are useful as a 24 h time stamp. When preparing the panel, rather than forming clear top and bottom layers, the unenriched sediment poured on top of the enriched sediment displaced the enriched sediment to the right and top. (The enriched sediment appears lighter in color than the unenriched sediment in the first frame of the video).
(MOV)

S2 Movie Detail of time-lapse series of Winogradsky panel with plaque-like pattern of clearing. Same movie as in S1 Movie but zoomed in on the left side of the panel and showing 48 days (2012/09/14–2012/11/01). Note a wave front sweep from the center of the left side moving up and to the right. Several other plaque-like regions are apparent at the bottom.
(MOV)

S3 Movie Detail of time-lapse series of Winogradsky panel with plaque-like pattern of clearing. Same movie as in S1 Movie but zoomed in on the top right side of the panel and showing 85 days (2012/09/12–2012/12/06). The movies shows initial abundant growth covering the black sediment, followed by clearing of the growth starting from central points that eventually merge.
(MOV)

S4 Movie Detail of time-lapse series of Winogradsky panel with plaque-like pattern of clearing. Same movie as in S1 Movie but zoomed in on the bottom-center of the panel and showing 48 days (2012/09/14–2012/11/01). Plaque-like zones are visible at the top right and bottom left.
(MOV)
