# Supplementary material for: Temporal and Spatial Distribution of the Microbial Community of Winogradsky Columns
Source: PLoS One. 2015 Aug 6;10(8):e0134588. doi: 10.1371/journal.pone.0134588 (PMC4527761; doi:10.1371/journal.pone.0134588)
Supplement: S1 Fig — 2D PCoA plots of unweighted UNIFRAC analysis showing first three principle component axes. A,B,C) Samples collected at indicated timepoints, colored by days of incubation. D,E,F) Samples collected at indicated timepoints, colored by location of sampling. G,H,I) Samples collected from surface or interior sections of Winogradsky columns incubated 60 days. J,K,L) Samples collected from columns incubated with or without light for 60 days. (PDF) [file pone.0134588.s001.pdf]

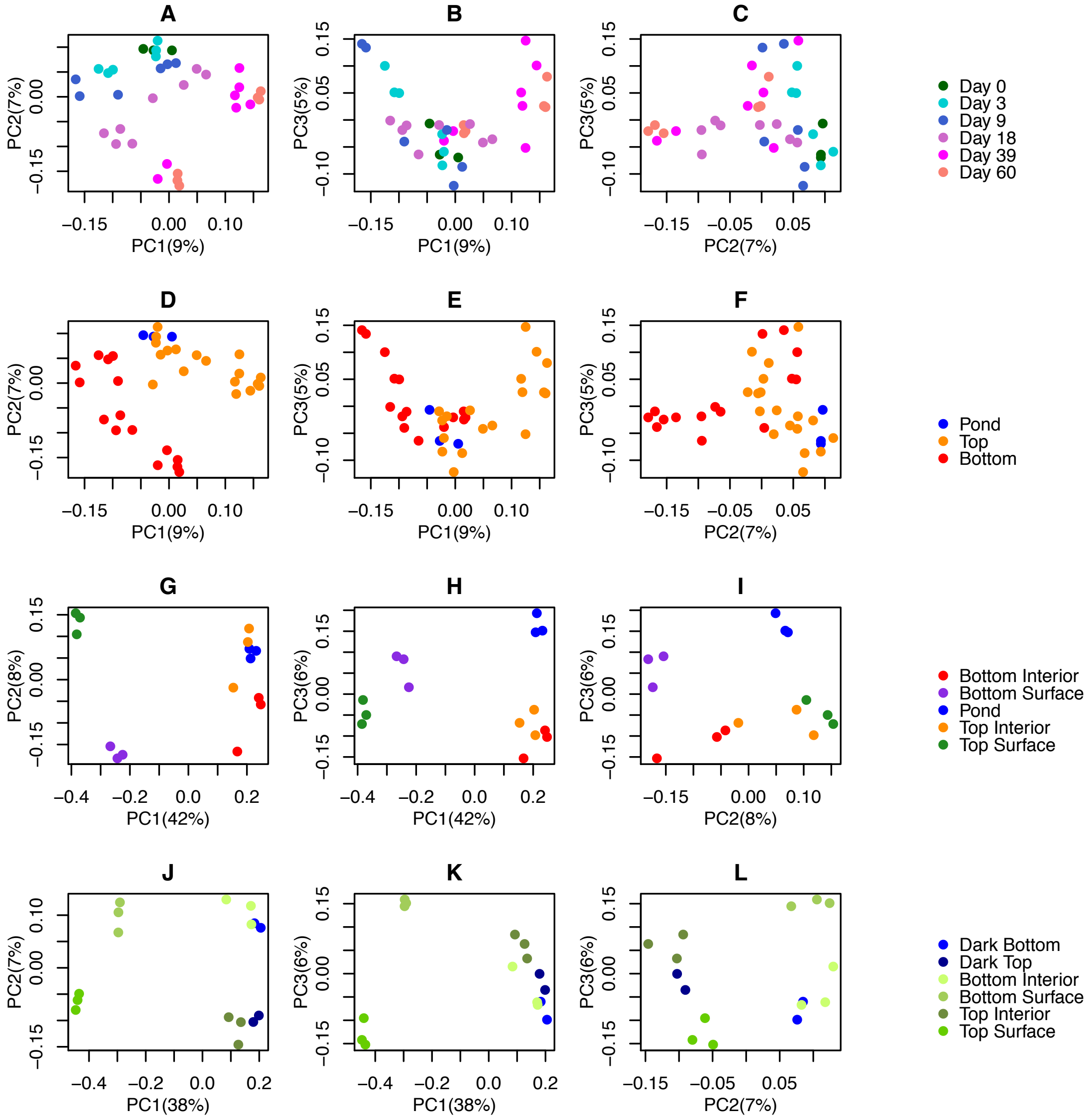

S2 Figure. Phylogenetic diversity in Winogradsky columns. 2D PCoA plots of unweighted UNIFRAC analysis showing first three principle component axes. A,B,C) Samples collected at indicated timepoints, colored by days of incubation. D,E,F) Samples collected at indicated timepoints, colored by location of sampling. G,H,I) Samples collected from surface or interior sections of Winogradsky columns incubated 60 days. J,K,L) Samples collected from columns incubated with or without light for 60 days.
